# Non‐neutralizing antibody responses following A(H1N1)pdm09 influenza vaccination with or without AS03 adjuvant system

**DOI:** 10.1111/irv.12780

**Published:** 2020-09-05

**Authors:** Damien Friel, Mary Co, Thierry Ollinger, Bruno Salaun, Anne Schuind, Ping Li, Karl Walravens, Francis A. Ennis, David W. Vaughn

**Affiliations:** ^1^ GSK Wavre Belgium; ^2^ University of Massachusetts Medical School Worcester MA USA; ^3^ GSK Rixensart Belgium; ^4^ GSK Rockville MD USA; ^5^ GSK King of Prussia PA USA; ^6^ Present address: Pfizer Collegeville PA USA; ^7^ Present address: Bill & Melinda Gates Foundation Seattle WA USA

**Keywords:** A(H1N1)pdm09 vaccine, AS03 adjuvant system, cross‐reactivity, non‐neutralizing antibodies

## Abstract

**Background:**

Non‐neutralizing antibodies inducing complement‐dependent lysis (CDL) and antibody‐dependent cell‐mediated cytotoxicity (ADCC) activity may contribute to protection against influenza infection. We investigated CDL and ADCC responses in healthy adults randomized to receive either non‐adjuvanted or AS03‐adjuvanted monovalent A(H1N1)pdm09 vaccine (containing 15 µg/3.75 μg of hemagglutinin, respectively) on a 2‐dose schedule 21 days apart.

**Methods:**

We conducted an exploratory analysis of a subset of 106 subjects having no prior history of A(H1N1)pdm09 infection or seasonal influenza vaccination enrolled in a previously reported study (NCT00985673). Antibody responses against the homologous A/California/7/2009 (H1N1) vaccine strain and a related A/Brisbane/59/2007 (H1N1) seasonal influenza strain were analyzed up to Day 42.

**Results:**

Baseline seropositivity determined with hemagglutination inhibition (HI), CDL and ADCC antibody titers against viral strains was high; A/California/7/2009 (HI [40.4‐48.1%]; CDL [34.6‐36.0%]; ADCC [92.1‐92.3%]); A/Brisbane/59/2007 (HI [73.1‐88.9%]; CDL [38.0‐42.0%]; ADCC [86.8‐97.0%]). CDL seropositivity increased following vaccination with both adjuvanted and non‐adjuvanted formulations (A/California/7/2009 [95.9‐100%]; A/Brisbane/59/2007 [75.5‐79.6%]). At Day 21, increases in CDL and ADCC antibody geometric mean titers against both strains were observed for both formulations. After 2 doses of AS03‐adjuvanted vaccine, vaccine responses of 95.8% (≥9‐fold increase from baseline in CDL titers) and 34.3% (≥16‐fold increase from baseline in ADCC titers) were seen against A/California/7/2009; and 22.4% and 42.9%, respectively, against A/Brisbane/59/2007. Vaccine responses after 2 doses of the non‐adjuvanted vaccine were broadly similar.

**Conclusions:**

Broadly comparable non‐neutralizing immune responses were observed following vaccination with non‐adjuvanted and AS03‐adjuvanted A(H1N1)pdm09 formulations; including activity against a related vaccine strain.

## 1 INTRODUCTION

Following influenza virus infection, a robust immune response is observed involving the generation of both neutralizing and non‐neutralizing antibodies.^1^ Neutralizing antibody responses are directed toward the viral hemagglutinin (HA) glycoprotein that mediates virus attachment to host cells via sialic acid receptor binding and subsequent cell entry. The importance of neutralizing HA‐antibodies in protection is well established, and influenza vaccines are developed and assessed primarily by their ability to induce hemagglutination inhibition (HI) as a surrogate for neutralizing antibodies.^2^, ^3^ However, most conventional HA‐specific neutralizing antibodies target epitopes of the HA globular head that, while immunodominant, are subject to substantial antigenic drift and are typically strain‐specific; hence the need for annual updating of the composition of the seasonal influenza vaccines so as to target and induce protective neutralizing antibodies to the anticipated predominant seasonal strains.^3^

Non‐neutralizing antibodies to influenza are also generated following infection and provide additional protection via a range of mechanisms including complement‐dependent lysis (CDL) and antibody‐dependent cell‐mediated cytotoxicity (ADCC).^4^, ^5^, ^6^, ^7^, ^8^, ^9^, ^10^, ^11^ Such non‐neutralizing antibodies can recognize and bind a range of viral epitopes expressed by influenza virus on the surface of infected cells with subsequent complement activation or direct cell lysis by natural killer (NK) cells, monocytes, and macrophages in conjunction with antiviral cytokine release.^6^, ^11^ A potential benefit of such non‐neutralizing antibodies is their recognition of epitopes within the HA globular head and also of more highly conserved epitopes (eg, in the HA stalk domain) than those recognized by neutralizing antibodies, so offering a broader cross‐reactive protection.^5^, ^12^ These non‐neutralizing antibodies include those directed against internal proteins such as nucleoprotein and matrix 1 protein to which ADCC antibody responses are observed following clinical infection or influenza vaccination.^9^, ^13^ These aspects are of particular relevance to pandemic influenza and associated vaccine development, where virus genomic reassortment events result in novel strains with novel viral epitopes in their more variable antigenic domains.^10^, ^11^, ^14^ In this respect, the role of CDL and ADCC antibodies in response to influenza infection or following vaccination is of considerable interest, with a number of reports in recent years.^7^, ^9^, ^13^, ^15^, ^16^, ^17^, ^18^ However, data from vaccine clinical studies are more limited.

Previously, we reported on a randomized controlled trial (NCT00985673) evaluating immunogenicity and safety of a monovalent A(H1N1)pdm09 pandemic influenza vaccine given with or without AS03 adjuvant.^19^ Use of adjuvants such as AS03 is an important consideration in pandemic vaccine development as it provides an antigen sparing component to vaccine composition, which may be relevant when antigen availability to novel strains is limited. In that study, robust HI antibody responses were observed with both the non‐adjuvanted (15 µg of hemagglutinin) and AS03‐adjuvanted vaccine formulations (3.75 µg of hemagglutinin); where the differences in hemagglutinin content in these different formulations represent such an antigen sparing effect.^19^ To investigate the effect of these vaccine formulations on non‐neutralizing antibody responses, we performed an exploratory evaluation and analysis of CDL and ADCC antibody responses, using sera collected in a sub‐population of this study cohort. The aims were to characterize non‐neutralizing antibody immunogenicity against the homologous A(H1N1)pdm09 vaccine strain (A/California/7/2009) and also against a related seasonal influenza strain; A/Brisbane/59/2007(H1N1) representative of a previously circulating seasonal H1N1 subtype (which in the context of the present analysis we consider this to be a heterologous vaccine strain).

## 2 MATERIALS AND METHODS

### 2.1 Study design and study population

This was an exploratory analysis of a sub‐population of a previously reported randomized, observer‐blind, controlled clinical trial (NCT00985673) conducted in the United States and Canada between October 2009 and December 2010. The study design, inclusion criteria, and primary objectives (immunogenicity and safety) have previously been published.^19^ Participants were healthy adults 19 to 40 years of age, excluding subjects with any prior history of A(H1N1)pdm09 influenza vaccination or physician‐confirmed A(H1N1)pdm09 infection, and those with a history of previous seasonal influenza vaccination.^19^ The study was conducted in accordance with Good Clinical Practice and the Declaration of Helsinki. All study‐related documents were approved by the appropriate Institutional Review Boards of participating Centres; and written informed consent was obtained from all subjects prior to enrollment. Anonymized individual participant data and study documents can be requested for further research from www.clinicalstudydatarequest.com.

The current analysis involved a subset of those participants from this parent study (corresponding to groups E and F) who were randomized to receive non‐adjuvanted or AS03‐adjuvanted formulations, respectively, of a A(H1N1)pdm09 pandemic influenza vaccine.^19^ The A(H1N1)pdm09 pandemic influenza vaccine is a monovalent, inactivated, split‐virion antigen either in non‐adjuvanted form (with 15 µg of hemagglutinin) or as an AS03‐adjuvanted formulation (*Arepanrix*, GSK, Belgium); with 3.75 µg of hemagglutinin,^19^ and which contains DL‐α‐tocopherol and squalene in an oil‐in‐water emulsion.^20^

Subjects in this exploratory analysis were randomly drawn from the according‐to‐protocol (ATP) cohort in each group, with subject selection based on available blood samples for additional testing for non‐neutralizing antibody responses. Subjects received either non‐adjuvanted or AS03‐adjuvanted formulations on Days 0 and 21 administered in the deltoid muscle; no other vaccines were administered during this time‐frame (and so for the purposes of the present analysis received only the monovalent vaccine).^19^

Blood samples were collected on Day 0 (pre‐vaccination) and on Days 21 and 42 (ie, 21 days after each vaccine dose). All samples were coded with a unique identification number, aliquoted, and stored at −80°C until analysis.

### 2.2 Immunogenicity assays

HI antibody titers against the homologous H1N1pdm09 vaccine strain (inactivated A/California/7/2009) and the seasonal influenza strain (A/Brisbane/59/2007) were measured (in duplicate) as previously reported in the primary study using a validated assay cut‐off value (≥1:10).^19^

CDL and ADCC assays were performed at the University of Massachusetts Medical School (UMMS). The principles and technical details of these chromium‐release assays have previously been described.^7^, ^15^ For the CDL assay, A549 cells (human lung epithelial cell line; ATCC CCL‐185) were infected with egg‐derived A/California/7/09 (H1N1)pdm09 and A/Brisbane/59/2007 virus strains incubated overnight then labeled with ^51^chromium for 1‐hour. These infected target cells were then seeded in 96‐well plates (at 2000 cells/well) to which 3‐fold serial dilutions (1:32‐1776) of heat‐inactivated sera from study subjects were added to appropriate wells (in replicates of three) and incubated in the presence of complement (Low‐Tox Guinea Pig Complement, Cedarlane Laboratories, Burlington, North Carolina). RENEX detergent or RPMI 1640 medium was added to maximum release and minimum release wells, respectively. Plates were centrifuged at 250 *g* for 5 minutes, pelleted cells incubated for 2‐hours, and then supernatants harvested from each well and counted in a gamma counter as counts per minute (CPM). In all assays, serum from an adult with CDL antibodies against seasonal A(H1N1) was used as a positive control. The percentage specific immune lysis (%SIL) of infected A549 cells was then calculated at each serum dilution as follows: %SIL = (CPM of experimental release−average CPM of complement minimum release)/(average CPM of maximum release−average CPM of complement minimum release)*100. The CDL serum antibody endpoint titer was defined as the highest serum dilution at which ≥50% peak SIL of the sample was observed. In previous studies, we have used different thresholds including ≥15%,^7^ and also ≥50%,^6^ as in the present study. Based upon this ≥50% peak SIL threshold, the assay cut‐off was established as 32.0 (first assay dilution [1/DIL]), and subjects with a titer <32.0 1/DIL were considered seronegative.

For the ADCC assay, A549 cells were infected with A/California/7/09 (H1N1)pdm09 and A/Brisbane/59/2007 virus strains, then ^51^chromium‐labeled and plated in a similar manner as in the CDL assay. Heat‐inactivated sera from study subjects were added in 4‐fold serial dilutions (1:32‐1:32 768) in the presence of enriched NK cells derived and sourced from peripheral blood mononuclear cell samples obtained from nine healthy subjects >18 years old at UMMS (with informed consent and Institutional Review Board approval). NK cells were added in an effector‐to‐target cell ratio of 5:1. RENEX detergent or RPMI medium was then added and supernatants subsequently harvested in a similar manner as for the CDL assay. All sera were tested in replicates of three and serum from an adult with ADCC antibodies against seasonal A(H1N1) used as a positive control. The %SIL of infected A549 cells was then calculated at each serum dilution as (% lysis of sample−% lysis of NK cells alone). Calculation of % lysis of sample or of NK cells alone was calculated as follows: % lysis of sample = (average CPM of experimental release−average CPM of minimum release)/(average CPM of maximum release−average CPM of minimum release)*100; % lysis of NK cells = (average CPM of NK cell release−average CPM of minimum release)/(average CPM of maximum release−average CPM of minimum release)*100. The ADCC antibody endpoint titer was defined in a similar manner as for the CDL assay, that is, the highest serum dilution at which ≥50% peak SIL of the sample was observed, with a similar cut‐off value (32.0 1/DIL) used.

### 2.3 Immunogenicity assessments

HI seropositivity status was assessed at baseline (Day 0) and on Day 42; seropositivity rate was defined as the percentage of subjects with HI titer equal to or above the assay cut‐off value (≥10), consistent with the approach used in the parent study and other studies evaluating HI immunogenicity in response to the H1N1pdm09 vaccine.^19^, ^21^

For both CDL and ADCC assays, we evaluated seropositivity, geometric mean titer (GMT), vaccine response (VR), and mean geometric increase (MGI). Subjects were considered seropositive for CDL or ADCC antibodies if their antibody titer was equal to or above the assay cut‐off (≥32.0 1/DIL). For GMT calculations, titers were log_10_ transformed and then calculations performed using the antilog of the mean of the log_10_ titer transformations. For CDL, titers <32.0 1/DIL were assigned a value of 10.7 1/DIL (to account for the 3‐fold dilution), and for endpoint titers above the maximum assay readout value of the serum dilutions tested (>7776 1/DIL), a maximal titer of 23 328 1/DIL was assigned. For ADCC, titers <32.0 1/DIL were assigned a value of 8.0 1/DIL (to account for the 4‐fold dilution); endpoint titers above the maximum assay readout (>32 768 1/DIL) were assigned a maximal titer of 131 072 1/DIL.

No standardized VR criteria exist, and to account for this uncertainty, we used two exploratory levels of response thresholds to determine VR, and applied both to assess VR at Day 21 and Day 42. For the CDL assay, VR was defined as the proportion of subjects who showed a 3‐fold or 9‐fold increase in the post‐vaccination reciprocal titer from baseline. For those subjects with baseline titers of <32.0 1/DIL (considered seronegative at baseline), post‐vaccination reciprocal titers of ≥96.0 1/DIL or ≥288.0 1/DIL were required to meet these 3‐fold and 9‐fold thresholds, respectively. For the ADCC assay, a similar approach was adopted (but using subsequent 4‐fold or 16‐fold increases from baseline in post‐vaccination reciprocal titers; subjects seronegative at Day 0 required post‐vaccination reciprocal titers of ≥128 1/DIL or ≥512.0 1/DIL to meet these thresholds). MGI was defined as the geometric mean fold rise in GMTs at Day 21/Day 42 relative to Day 0.

### 2.4 Data analysis

Descriptive analyses were performed for all data and summarized for each time‐point for each study group (ie, subjects receiving either the non‐adjuvanted or AS03‐adjuvanted vaccine). GMTs, MGIs, and the percentage of seropositive subjects, and VRs (with 95% confidence intervals [CIs]) were each tabulated. Reverse cumulative distribution curves at Day 42 were also generated. For each of the CDL and ADCC datasets, differences between groups (ie, AS03‐adjuvanted vaccine group minus non‐adjuvanted vaccine group) in the percentage of subjects with a VR at Day 42 were calculated. The GMT ratio at Day 42 was analyzed via analysis of covariance (ANCOVA) models on the log_10_ transformed titers, including the vaccine group as a fixed effect and age and the baseline value (Day 0) as covariates. The 95% CI for the adjusted‐GMT ratio was obtained by exponential‐transformation of 95% CIs for the mean of log‐transformed titer. Correlations between HI, CDL, and ADCC antibody titers were evaluated using linear regression and by calculating the correlation coefficient (*r*) values. Correlation scores were considered as follows: <0.63 as poor, 0.63‐0.77 as fair, 0.77‐0.86 as good, and >0.86 as excellent.

All statistical analyses were performed using the SAS software (SAS Institute Inc, NC, United States).

## 3 RESULTS

**Figure 1:**
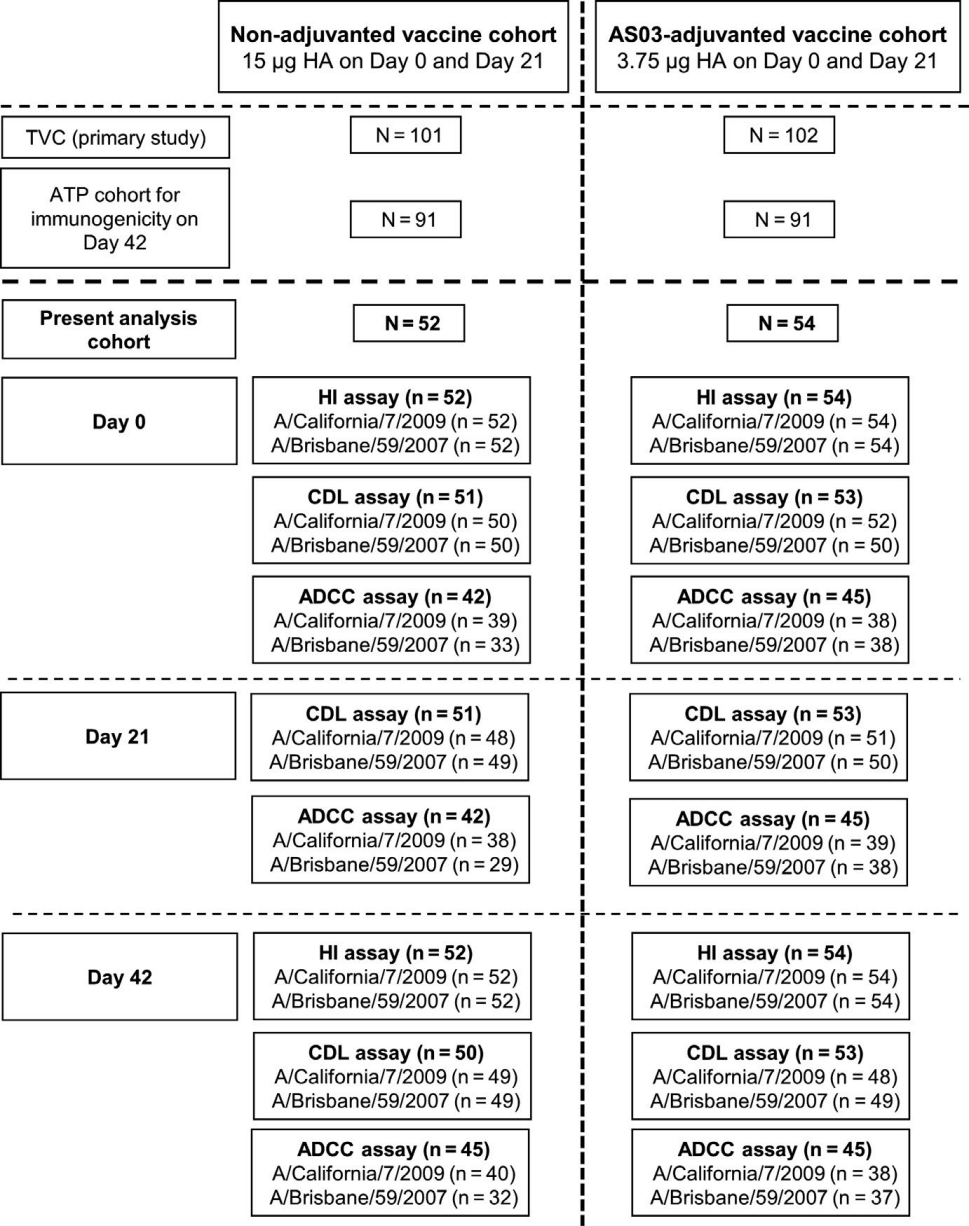
Study flow. From the original according‐to‐protocol cohort from the primary study, 106 subjects were included: 52 receiving the non‐adjuvanted vaccine and 54 receiving the AS03‐adjuvanted vaccine. ADCC, antibody‐dependent cell‐mediated cytotoxicity; ATP, according‐to‐protocol; CDL, complement‐dependent lysis; HA, hemagglutinin; HI, hemagglutinin inhibition; n, number of subjects with available results for all three antibody responses (HI, CDL and ADCC); N, total number of subjects in the non‐adjuvanted or AS03‐adjuvanted vaccine group; TVC, total vaccinated cohort

**Figure 2:**
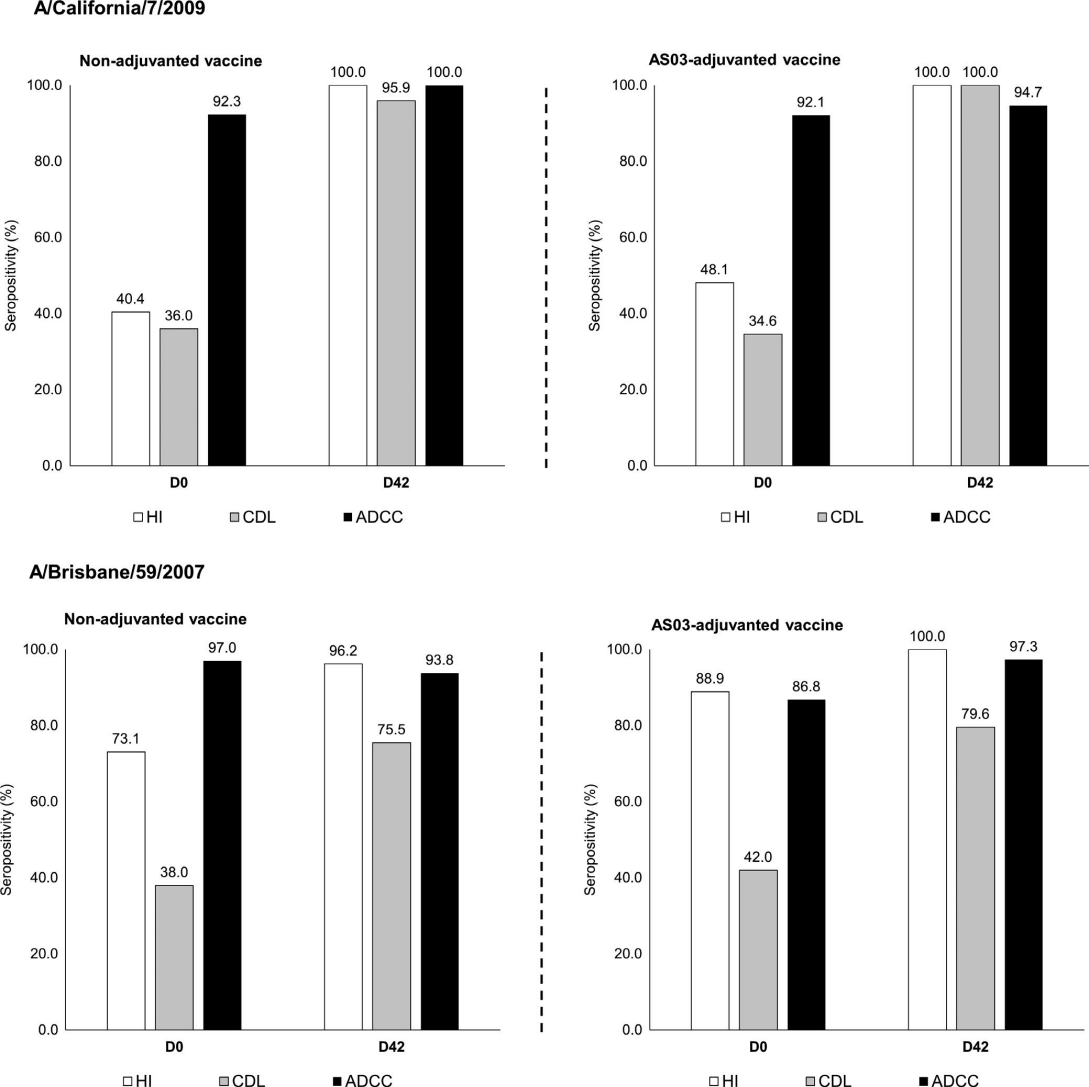
Baseline seropositivity to hemagglutination inhibition (HI), complement‐dependent lysis (CDL), and antibody‐dependent cell‐mediated cytotoxicity (ADCC) antibodies against A/California/7/2009 and A/Brisbane/59/2007 strains at baseline (Day 0) and Day 42. D, Day

From the original ATP cohort from the primary study, 106 subjects were included in this exploratory analysis (accounting for approximately 50% of eligible participants); 52 receiving the non‐adjuvanted vaccine and 54 receiving the AS03‐adjuvanted vaccine. Study flow and number of subjects assayed at study time‐points are shown in Figure 1. Cohort demographics are presented in Table 1. Baseline HI antibody titers indicated that 40.4‐48.1% of subjects were seropositive for the A/California/7/2009 strain and 73.1‐88.9% for the A/Brisbane/59/2007 strain (Table 1 and Figure 2).

**Table 1:** Sociodemographic parameters for present analysis according‐to‐protocol cohort for immunogenicity

Patient characteristics	Non‐adjuvanted vaccine N = 52	AS03‐adjuvanted vaccine N = 54
Age, years		
Mean (SD)	29.5 (6.3)	28.9 (6.6)
Range	19‐40	19‐40
Gender, n (%)		
Female	36 (69.2%)	32 (59.3%)
Male	16 (30.8%)	22 (40.7%)
Evidence of previous natural infection^a^, n (%)		
A/California/7/2009 strain	21 (40.4%)	26 (48.1%)
A/Brisbane/59/2007 strain	38 (73.1%)	48 (88.9%)

Abbreviations: AS03, Adjuvant System containing DL‐α‐tocopherol and squalene in an oil‐in‐water emulsion; n, number of subjects with available results; N, total number of subjects in the non‐adjuvanted or AS03‐adjuvanted vaccine groups; SD, standard deviation.

^a^ Based upon baseline hemagglutination inhibiting antibody measurements (titers ≥ 10 at Day 0).

### 3.1 Immunogenicity based on CDL antibody assay

Prior to vaccination, baseline seropositivity for CDL antibodies for A/California/7/2009 in each study group ranged between 34.6% and 36.0%, and between 38.0% and 42.0% for A/Brisbane/59/2007 (Table 2 and Figure 2). Twenty‐one days after vaccination with the first dose of non‐adjuvanted vaccine, seropositivity increased to 95.8% against A/California/7/2009 and 67.3% against A/Brisbane/59/2007; all subjects receiving the AS03‐adjuvanted vaccine were seropositive against A/California/7/2009 and 76.0% seropositive against A/Brisbane/59/2007. At Day 42, the high proportion of subjects seropositive against A/California/7/2009 was maintained for both vaccine formulations, with an increase in the proportion of participants seropositive against the A/Brisbane/59/2007 strain also observed for both vaccine groups (Table 2 and Figure 2).

**Table 2:** Complement‐dependent lysis (CDL) and antibody‐dependent cell‐mediated cytotoxicity (ADCC) antibody responses (seropositivity and geometric mean titers [GMTs]) to A/California/7/2009 and A/Brisbane/59/2007 strains (according‐to‐protocol cohort for immunogenicity)

	Non‐adjuvanted vaccine (N = 52)			AS03‐adjuvanted vaccine (N = 54)		
	n/N^a^	Seropositive % (95% CIs)	GMT (95% CIs)	n/N^a^	Seropositive % (95% CIs)	GMT (95% CIs)
CDL antibodies						
A/California/7/2009 strain						
Day 0	18/50	36.0 (22.9‐50.8)	26.7 (17.9‐40.0)	18/52	34.6 (22.0‐49.1)	24.5 (17.1‐35.1)
Day 21	46/48	95.8 (85.7‐99.5)	1331.4 (788.7‐2247.8)	51/51	100 (93.0‐100)	2940.6 (2067.2‐4182.8)
Day 42	47/49	95.9 (86.0‐99.5)	1651.9 (1007.9‐2707.6)	48/48	100 (92.6‐100)	5421.9 (3951.3‐7439.9**)**
A/Brisbane/59/2007 strain						
Day 0	19/50	38.0 (24.7‐52.8)	30.1 (19.9‐45.4)	21/50	42.0 (28.2‐56.8)	31.8 (20.7‐49.1)
Day 21	33/49	67.3 (52.5‐80.1)	72.1 (44.9‐115.6)	38/50	76.0 (61.8‐86.9)	160.1 (93.0‐275.8)
Day 42	37/49	75.5 (61.1‐86.7)	127.2 (78.2‐206.9)	39/49	79.6 (65.7‐89.8)	179.1 (106.7‐300.7)
ADCC antibodies						
A/California/7/2009 strain						
Day 0	36/39	92.3 (79.1‐98.4)	1202.5 (568.3‐2544.2)	35/38	92.1 (78.6‐98.3)	970.4 (499.0‐1887.1)
Day 21	35/38	92.1 (78.6‐98.3)	6243.8 (2605.8‐14 961.1)	36/39	92.3 (79.1‐98.4)	6991.4 (2837.4‐17 226.8)
Day 42	40/40	100 (91.2‐100)	12 403.6 (6321.2‐24 338.5)	36/38	94.7 (82.3‐99.4)	9533.0 (4180.0‐21 741.4)
A/Brisbane/59/2007 strain						
Day 0	32/33	97.0 (84.2‐99.9)	1185.9 (598.1‐2351.1)	33/38	86.8 (71.9‐95.6)	758.3 (327.1‐1757.9)
Day 21	28/29	96.6 (82.2‐99.9)	8994.1 (3856.5‐20 976.1)	37/38	97.4 (86.2‐99.9)	9978.7 (4789.7‐20 789.4)
Day 42	30/32	93.8 (79.2‐99.2)	6876.0 (2718.2‐17 393.7)	36/37	97.3 (85.8‐99.9)	9822.0 (4530.1‐21 295.8)

Abbreviations: ADCC, antibody‐dependent cell‐mediated cytotoxicity; AS03, Adjuvant System containing DL‐α‐tocopherol and squalene in an oil‐in‐water emulsion; CDL, Complement‐dependent lysis; CIs, confidence intervals; GMT, geometric mean titer.

^a^ n, number of (seropositive) subjects with antibody titer ≥ 32.0 1/dilution on CDL or ADCC assay; N, number of subjects with available results.

**Figure 3:**
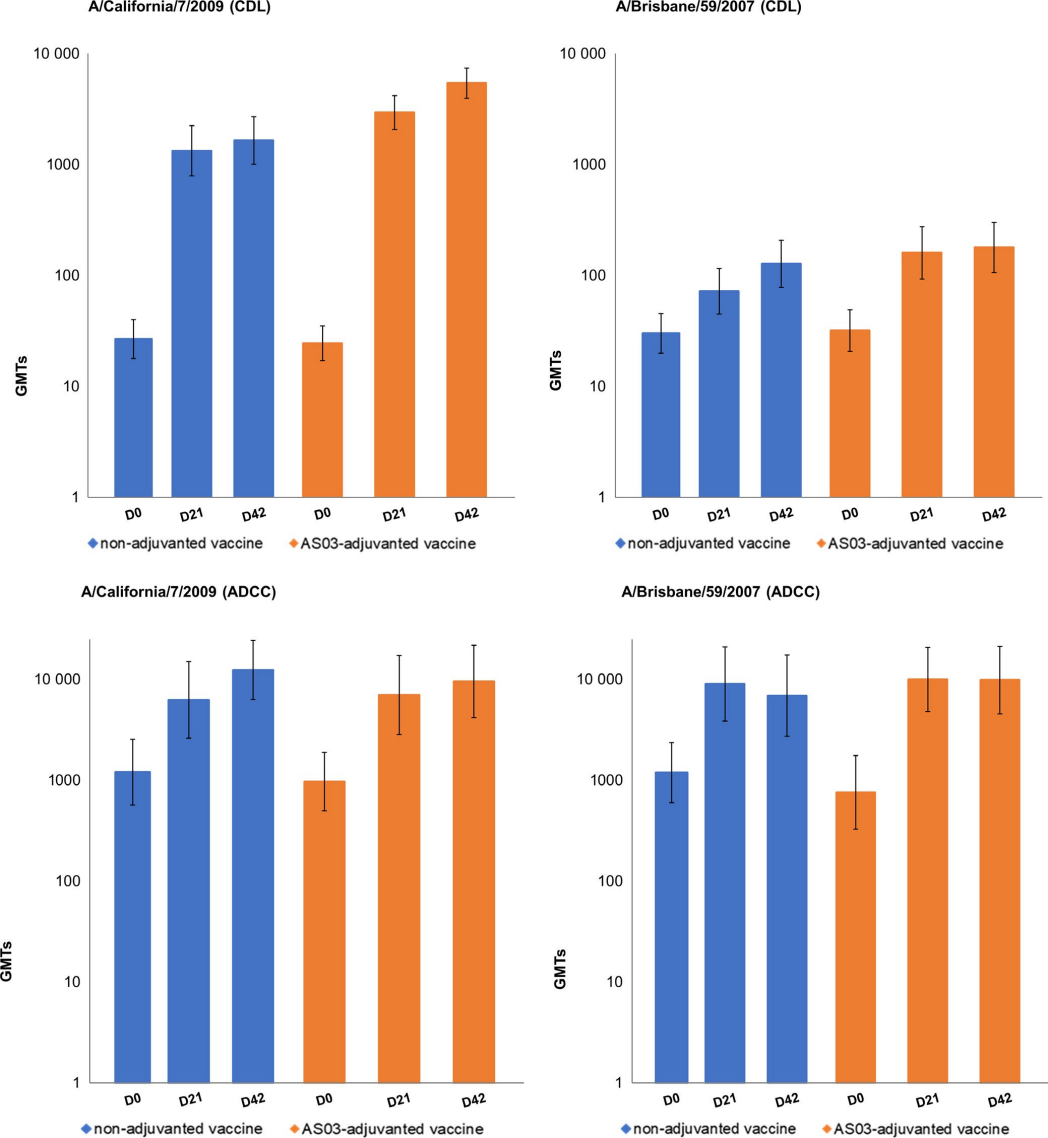
CDL and ADCC antibody geometric mean titers (GMTs) against A/California/7/2009 and A/Brisbane/59/2007 strains at baseline and Days 21 and 42. Upper panel represents GMTs as measured by CDL assay and lower panel by ADCC assay. ADCC, antibody‐dependent cell‐mediated cytotoxicity; CDL, complement‐dependent lysis; D, Day; GMT, geometric mean titer

Baseline CDL antibody GMTs in each study group ranged between 24.5 and 26.7 for A/California/7/2009 and between 30.1 and 31.8 for A/Brisbane/59/2007. Strong increases in GMTs were observed at 21 days post‐vaccination for either vaccine (Table 2 and Figure 3). The highest GMTs were observed against A/California/7/2009, with titers increasing with each vaccine dose; GMTs against the A/Brisbane/59/2007 strain also increased after each vaccine dose (Table 2). Reverse cumulative distribution curves at Day 42 demonstrating CDL and ADCC antibody responses against the homologous A/California/7/2009 vaccine strain and against A/Brisbane/59/2007 are shown in Figure S1.

**Figure 4:**
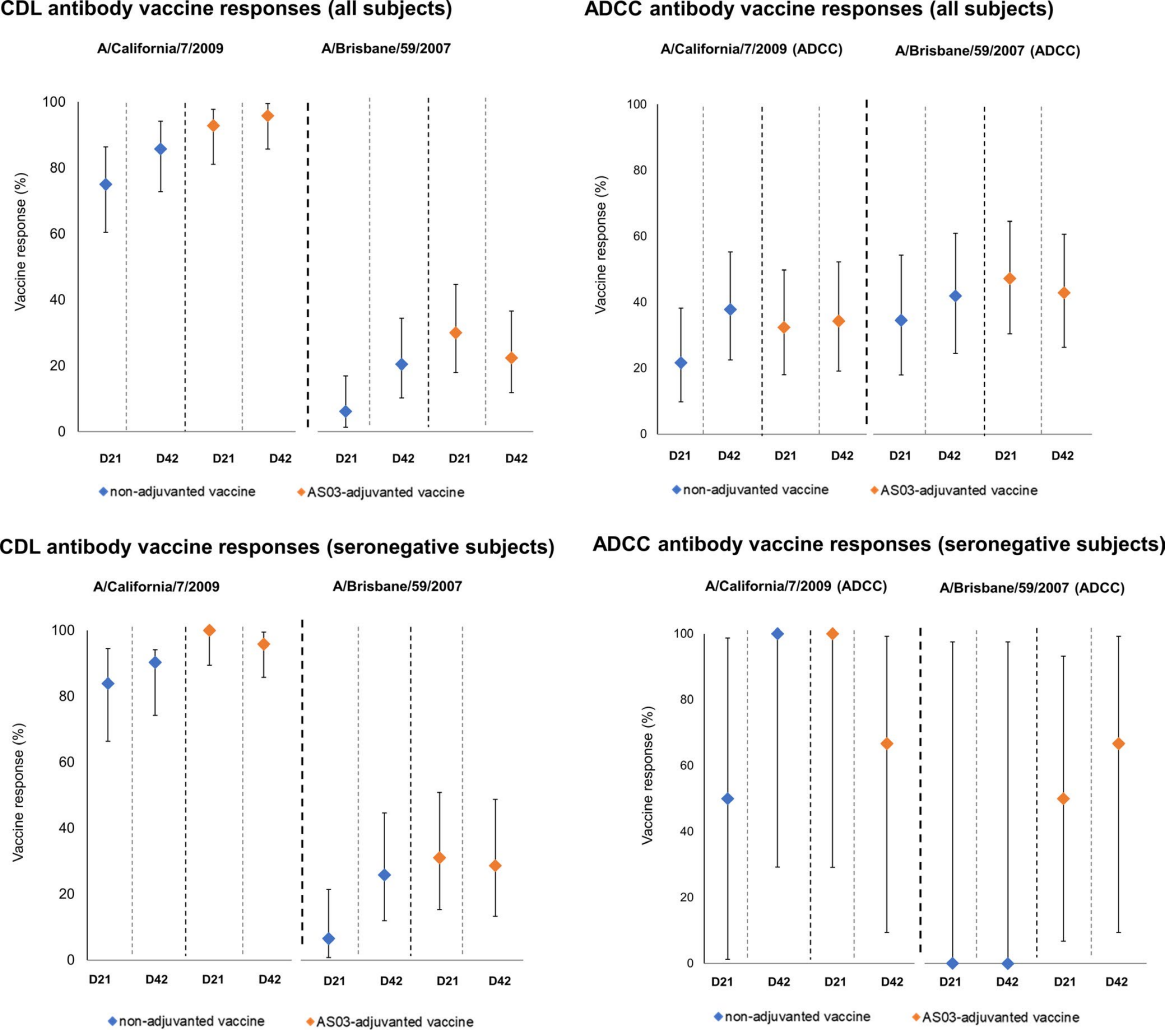
Vaccine responses in subjects receiving non‐adjuvanted and AS03‐adjuvanted vaccines against A/California/7/2009 and A/Brisbane/59/2007 strains at Day 21 and Day 42. Presented here are higher vaccine response thresholds; defined as a 9‐fold increase from baseline (Day 0) at Day 21 or Day 42 for CDL antibodies (and as a 16‐fold increase for ADCC antibodies); see also data in Table S2. ADCC, antibody‐dependent cell‐mediated cytotoxicity; CDL, complement‐dependent lysis; D, Day

CDL antibody VRs are shown in Figure 4 and Table S1. The great majority of subjects receiving either vaccine showed responses to the homologous A/California/7/2009 virus antigen after the first dose (Day 21) with over 90% of subjects in the non‐adjuvanted vaccine group and 100% of those receiving the AS03‐adjuvanted vaccine showing a 3‐fold increase in GMTs from baseline; the percentage of subjects with a 9‐fold increase in CDL titers from baseline was also high. These responses were maintained after the second dose (Day 42), where 9‐fold increases were observed in 85.7% of subjects in the non‐adjuvanted vaccine group and 95.8% of those receiving the AS03‐adjuvanted vaccine. For the A/Brisbane/59/2007 strain, VRs at Day 42, as assessed using the 9‐fold increase threshold, were observed in 20.4% (non‐adjuvanted vaccine group) and 22.4% (AS03‐adjuvanted vaccine) of subjects. For both strains, VRs were seen in a higher proportion of subjects seronegative at baseline than in seropositive subjects (Figure 4 and Table S1).

Differences in VRs between the AS03‐adjuvanted vaccine and the non‐adjuvanted groups are presented in Table S2 and adjusted GMTs, MGI and adjusted‐GMT ratio in Table S3. Between‐group differences in CDL VRs and adjusted‐GMT ratios against either vaccine strain were inconsistent, and with overlapping CIs in between‐group comparisons.

We evaluated correlations between CDL and HI antibody titers at Day 42, in both vaccine groups and in the total exploratory analysis cohort (Table S4 and Figure S2). Overall, there was fair linear correlation between HI and CDL titers against A/California/7/2009, with correlation coefficient (*r*) values of 0.68‐0.69 (Figure S2). Correlations between HI and CDL antibody titers against A/Brisbane/59/2007 were strong on initial analysis but after removal of an outlier patient the adjusted correlation was very poor, with *r* between 0.14 and 0.26 (Table S4).

### 3.2 Immunogenicity based on ADCC antibody assay

A high proportion of subjects in either study group were seropositive for ADCC antibodies against both vaccine strains at baseline, where seropositivity for A/California/7/2009 ranged between 92.1% and 92.3% and for A/Brisbane/59/2007 between 86.8% and 97.0% (Figure 2 and Table 2). At Day 42, 21 days after a second dose, all subjects receiving the non‐adjuvanted vaccine, and 94.7% of the AS03‐adjuvanted vaccine group were seropositive for ADCC antibodies against A/California/7/2009. For the A/Brisbane/59/2007 strain, seropositivity remained high following vaccination in subjects in the non‐adjuvanted group, while seropositivity increased in subjects receiving the AS03‐adjuvanted vaccine after the first dose (to 97.4%) with no subsequent change with the second dose.

ADCC antibody GMTs at baseline ranged from 970.4 to 1202.5 against A/California/7/2009 and from 758.3 to 1185.9 against A/Brisbane/59/2007. GMTs against A/California/7/2009 increased after each vaccine dose in both study groups; for A/Brisbane/59/2007 GMTs rose substantially after dose 1 then remained stable (AS03‐adjuvanted vaccine group) or declined (non‐adjuvanted group) (Table 2 and Figure 3). In contrast to GMTs observed with the CDL assay, GMT levels against A/Brisbane/59/2007 were of a broadly comparable level to those observed for A/California/7/2009 (Table 2 and Figure 3).

When evaluating ADCC responses, lower VRs against A/California/7/2009 virus antigen were seen compared to A/Brisbane/59/2007. For A/California/7/2009, the proportion of participants with a 16‐fold increase in ADCC titers from baseline was <40% in either group at Day 42; VRs against the A/Brisbane/59/2007 strain were marginally higher in both vaccine groups (41.9‐42.9) at this time‐point (Figure 4 and Table S1).

Similar to our CDL data, between‐group differences in VRs and adjusted‐GMT ratios for ADCC antibodies against either strain were inconsistent (Tables S2 and S3). Correlations between ADCC and HI antibody titers against A/California/7/2009 and against A/Brisbane/59/2007 at Day 42 were poor (Table S4). In addition, correlations between CDL and ADCC titers were also poor for either strain.

## 4 DISCUSSION

We evaluated CDL and ADCC antibody responses after immunization with either non‐adjuvanted or AS03‐adjuvanted pandemic A(H1N1)pdm09 vaccine in a subset of participants from a previously reported clinical trial,^19^ measuring antibody GMTs and VRs against the homologous (A/California/7/2009) strain and against a seasonal influenza heterologous strain (A/Brisbane/59/2007).

In this study cohort, a high proportion of subjects had HI antibody baseline titers to A/California/7/2009 (40‐48%) and A/Brisbane/59/2007 (73‐89%). We found that the numbers of subjects with detectable baseline CDL and ADCC antibodies to these A/California/7/2009 and A/Brisbane/59/2007 vaccine strains were also high. As measured via CDL antibodies, 35‐36% were seropositive against A/California/7/2009 and 38‐42% against A/Brisbane/59/2007. Baseline seropositivity for ADCC titers was substantially higher, with 92% of participants seropositive for the A/California/7/2009 strain and 87‐97% for A/Brisbane/59/2007.

As study participants had no prior history of trivalent inactivated influenza vaccine immunization or pandemic A(H1N1)pdm09 infection, such high pre‐vaccination seropositivity may reflect previous exposure (ie, subclinical natural infection to these vaccine strains). While past exposure to seasonal influenza may be expected, and so may explain the high seropositivity against A/Brisbane/59/2007, the high baseline seropositivity for the pandemic A/California/7/2009 strain is perhaps more surprising (as previous pandemic infection, although possible, may be considered less likely). A plausible explanation is that these pre‐vaccination levels represent pre‐existing cross‐reactive A(H1N1)pdm09‐specific ADCC antibodies (and to a lesser extent CDL antibodies) induced by previous seasonal influenza infection, rather than due to previous pandemic influenza infection. This is supported by data from previous studies where healthy adults had high levels of ADCC antibodies to pandemic A(H1N1) and A(H5N1) and A(H7N9) virus strains, even though it was considered unlikely that previous clinical exposure had occurred.^9^, ^13^, ^15^, ^16^ In this context, although we observed lower baseline with the CDL assay, this is consistent with previous data in which CDL antibodies against pandemic strains were detected in only a fraction of subjects with high ADDC antibody titers,^7^, ^15^ although different assay sensitivities may also have influenced our results.

Immunization with either unadjuvanted or AS03‐adjuvanted A(H1N1)pdm09 influenza vaccine was followed by increasing seropositivity rates and substantial increases in GMTs (for both CDL and ADCC antibodies) against both A/California/7/2009 and A/Brisbane/59/2007 strains. Only relatively minor (and non‐significant) differences in the kinetics and magnitude of responses were observed between the different vaccine formulations.

For CDL antibodies, GMTs against A/California/7/2009 and A/Brisbane/59/2007 following both the first and the second vaccine dose were higher in the AS03‐adjuvanted vaccine group than in non‐adjuvanted vaccine recipients. In addition, the kinetics of the CDL antibody response against A/California/7/2009 shows that while only modest increases in GMT were seen following a second dose of non‐adjuvanted vaccine, GMTs markedly increased following dose 2 of the AS03‐adjuvanted vaccine. ADCC antibody GMTs against A/California/7/2009 increased after each vaccine dose in both study groups. In contrast, while notable increases in GMTs for the heterologous A/Brisbane/59/2007 strain were observed following the first vaccine dose of either vaccine, a subsequent second dose resulted in little change. The more striking differences were observed for the VRs observed against different vaccine strains and as measured by different assays, with few differences between non‐adjuvanted and AS03‐adjuvanted vaccine groups. For the CDL assay, VRs were substantially higher against the homologous A/California/7/2009 strain than for the heterologous A/Brisbane/59/2007 strain. In contrast, for ADCC responses, VRs were relatively similar against either strain. Looking across these response data, we see that VRs against the A/California/7/2009 strain were far higher when evaluating CDL antibodies than those responses seen with ADCC antibodies; in contrast, for the A/Brisbane/59/2007 strain, the higher responses were observed when using the ADCC assay. We have no obvious explanation for this, and in part it may reflect the uncertainty around the response thresholds we used.

The generally comparable immunogenicity we observed with the AS03‐adjuvanted vaccine in generating robust CDL and ADCC responses is consistent with that seen for HI responses in the primary study,^19^ as well as that from other studies evaluating conventional HI immunogenicity of AS03‐adjuvanted pandemic A(H1N1)pdm09 vaccines. In the latter, lower HA antigen induces comparable HI antibody responses to conventional non‐adjuvanted formulations with higher HA content.^21^, ^22^ Our results are also consistent with other recent data on AS03‐adjuvanted H1N1pdm09 vaccine, where robust ADCC responses were observed in subjects regardless of baseline HI seropositivity.^17^, ^18^ They are also consistent with studies using other influenzas vaccine, including a recent study demonstrating ADCC responses to quadrivalent and MF‐59 adjuvanted vaccines in older adults.^23^

The present study has some limitations. While we have evaluated responses at a group level, we have not accounted for individual subject responses, and the impact that baseline seropositivity (in the context of primed and unprimed subjects) may have had in subsequent CDL and ADCC antibody responses. Data suggest that while post‐vaccination titers for HI and ADCC antibodies are broadly comparable in primed and unprimed subjects, the vaccine responses in terms of foldincreases from baseline may differ; it has been reported that higher fold increases in HI and ADCC titers in response to AS03‐adjuvanted A(H1N1)pdm09 vaccine are seen in unprimed subjects.^17^ The CDL and ADCC assays we used, while developed and qualified in a similar manner as in previous studies,^7^, ^13^, ^15^ were exploratory, as were our vaccine response thresholds. For ADCC responses, NK cells were sourced from multiple healthy donors, and donor variation in NK cells may have influenced our ADCC responses. Another limitation is that we cannot identify the viral epitopes which determine the pre‐existing CDL and ADCC antibody titers and subsequent responses.

## 5 CONCLUSIONS

Cross‐reactive CDL and ADCC antibodies may constitute crucial components of immune responses and provide some level of protection against existing and emerging pandemic influenza viruses.^24^ Their role in providing protection against seasonal influenza is increasingly recognized, as is the need to more fully consider non‐neutralizing antibody responses in vaccine development for a better characterization of the immune response of next‐generation vaccine candidates.^25^

Our data support these views and provide further evidence that ADCC and CDL assays should be an important consideration in vaccine development and evaluation and for the design of future, more cross‐reactive vaccines (eg, universal vaccine). The possibility that antigens in the vaccine such as nucleoprotein and matrix 1 protein might contribute to cross‐reactive, non‐neutralizing immune responses cannot be ruled out. Determining the levels of these antigens in the vaccine would be important to investigate this. Non‐neutralizing immune responses following vaccination with non‐adjuvanted and AS03‐adjuvanted formulations were observed; responses were broadly comparable with either vaccine formulation. Seropositivity, GMTs and vaccine responses for CDL antibodies were greater against the homologous A/California/7/2009 strain than for the A/Brisbane/59/2007 strain, but there was substantial evidence of cross‐reactivity. For ADCC antibodies, more variability was observed, and there were higher VRs toward the heterologous A/Brisbane/59/2007 strain. Reduced HA antigen content (antigen sparing) in the AS03‐adjuvanted formulation did not impact upon the magnitude or kinetics of these responses.

## 6 TRADEMARKS

*Arepanrix* is a trademark owned by or licensed to the GSK group of companies.

## CONFLICT OF INTEREST

DF, TO, BS, AS and KW are employed by the GSK group of companies. PL and DV were employees of the GSK group of companies during the conduct of the study. BS, AS, KW and PL hold shares from the GSK group of companies. MC and FE received funds from GSK through their institution for the conduct of the study. None of the authors declare non‐financial conflicts of interest.

## AUTHOR CONTRIBUTION

**Damien Friel:** Conceptualization (equal); Data curation (equal); Formal analysis (equal); Methodology (equal); Writing‐review & editing (equal). **Mary Co:** Conceptualization (equal); Data curation (equal); Formal analysis (equal); Methodology (equal); Writing‐review & editing (equal). **Thierry Ollinger:** Conceptualization (equal); Data curation (equal); Formal analysis (equal); Methodology (equal); Writing‐review & editing (equal). **Bruno Salaun:** Formal analysis (equal); Writing‐review & editing (equal). **Anne Schuind:** Formal analysis (equal); Investigation (equal); Writing‐review & editing (equal). **Ping Li:** Conceptualization (equal); Data curation (equal); Formal analysis (equal); Writing‐review & editing (equal). **Karl Walravens:** Conceptualization (equal); Formal analysis (equal); Writing‐review & editing (equal). **Francis A Ennis:** Conceptualization (equal); Data curation (equal); Investigation (equal); Methodology (equal); Writing‐review & editing (equal). **David W Vaughn:** Conceptualization (equal); Formal analysis (equal); Writing‐review & editing (equal).

All authors participated in the design or implementation or analysis, and in study interpretation and manuscript development of this manuscript. All authors had full access to the data and gave final approval before submission.

## Supporting information

Supplementary Material
