# Supplementary material for: Non‐neutralizing antibody responses following A(H1N1)pdm09 influenza vaccination with or without AS03 adjuvant system
Source: Influenza Other Respir Viruses. 2020 Sep 5;15(1):110–20. doi: 10.1111/irv.12780 (PMC7767944; doi:10.1111/irv.12780)
Supplement: Supplementary file 1 — Supplementary Material [file IRV-15-110-s001.docx]

**Supplementary Information**

**Non-neutralizing antibody responses following A(H1N1)pdm09 influenza vaccination with or without AS03 adjuvant system**

**Authors:** Damien Friel,^1^ Mary Co,^2^ Thierry Ollinger,^1^ Bruno Salaun,^3^ Anne Schuind,^4^ Ping Li,^5^ Karl Walravens,^1^ Francis A. Ennis,^2^ David W. Vaughn^4^

**Affiliations:**

^1^ GSK, Wavre, Belgium

^2^ University of Massachusetts Medical School, Worcester, MA, USA

^3^ GSK, Rixensart, Belgium

^4^ GSK, Rockville, MD, USA

^5^ GSK, King of Prussia, PA, USA

**Corresponding author:** Damien Friel, GSK, Avenue Fleming 20, 1300 Wavre, Belgium

[**SUPPLEMENTARY TABLE 1** Vaccine responses to vaccine strains (according-to-protocol cohort for immunogenicity) 2](#_Toc39445166)

[**SUPPLEMENTARY TABLE 2.** Study group vaccine response (VR) comparisons (according-to-protocol cohort for immunogenicity) 5](#_Toc39445167)

[**SUPPLEMENTARY TABLE 3.** Mean geometric increases (MGIs), adjusted geometric mean titers (GMTs) and adjusted-group ratios (according-to-protocol cohort for immunogenicity). 6](#_Toc39445168)

[**SUPPLEMENTARY TABLE 4** Hemagglutination inhibition (HI), complement-dependent lysis (CDL), and antibody-dependent cell-mediated cytotoxicity (ADCC) antibody titer correlations at Day 42 (according to protocol cohort for immunogenicity). 7](#_Toc39445169)

[**FIGURE S1** Reverse cumulative distribution curves for antibody responses against influenza strains (according-to-protocol cohort for immunogenicity). 8](#_Toc39445170)

[**FIGURE S2** Correlations between hemagglutination inhibition (HI) and complement-dependent lysis (CDL) antibody titers against A/California/7/2009 vaccine strain at Day 42. 9](#_Toc39445171)

**SUPPLEMENTARY TABLE 1** Vaccine responses to vaccine strains (according-to-protocol cohort for immunogenicity)

|  | **Non-adjuvanted vaccine (N=52)** | | | | **AS03-adjuvanted vaccine (N=54)** | | | |
| --- | --- | --- | --- | --- | --- | --- | --- | --- |
|  | **Vaccine response ^a,b^** | | | | **Vaccine response ^a,b^** | | | |
| **CDL antibodies** | (3-fold increase) | | (9-fold increase) | | (3-fold increase) | | (9-fold increase) | |
|  | n/N^c^ | % (95% CIs) | n/N^c^ | % (95% CIs) | n/N^c^ | % (95% CIs) | n/N^c^ | % (95% CIs) |
| **A/California/7/2009 strain** |  |  |  |  |  |  |  |  |
| Day 21 |  |  |  |  |  |  |  |  |
| Seronegative | 28/31 | 90.3 (74.2–98.0) | 26/31 | 83.9 (66.3–94.5) | 33/33 | 100 (89.4–100) | 33/33 | 100 (89.4–100) |
| Seropositive | 16/17 | 94.1 (71.3–99.9) | 10/17 | 58.8 (32.9–81.6) | 18/18 | 100 (81.5–100) | 14/18 | 77.8 (52.4–93.6) |
| All | 44/48 | 91.7 (80.0–97.7) | 36/48 | 75.0 (60.4–86.4) | 51/51 | 100 (93.0–100) | 47/51 | 92.2 (81.1–97.8) |
| Day 42 |  |  |  |  |  |  |  |  |
| Seronegative | 28/31 | 90.3 (74.2–98.0) | 28/31 | 90.3 (74.2–98.0) | 31/31 | 100 (88.8–100) | 31/31 | 100 (88.8–100) |
| Seropositive | 16/18 | 88.9 (65.3–98.6) | 14/18 | 77.8 (52.4–93.6) | 17/17 | 100 (80.5–100) | 15/17 | 88.2 (63.6–98.5) |
| All | 44/49 | 89.8 (77.8–96.6) | 42/49 | 85.7 (72.8–94.1) | 48/48 | 100 (92.6–100) | 46/48 | 95.8 (85.7–99.5) |
| **A/Brisbane/59/2007 strain** |  |  |  |  |  |  |  |  |
| Day 21 |  |  |  |  |  |  |  |  |
| Seronegative | 13/31 | 41.9 (24.5–60.9) | 2/31 | 6.5 (0.8–21.4) | 16/29 | 55.2 (35.7–73.6) | 9/29 | 31.0 (15.3–50.8) |
| Seropositive | 3/18 | 16.7 (3.6–41.4) | 1/18 | 5.6 (0.1–27.3) | 11/21 | 52.4 (29.8–74.3) | 6/21 | 28.6 (11.3–52.2) |
| All | 16/49 | 32.7 (19.9–47.5) | 3/49 | 6.1 (1.3–16.9) | 27/50 | 54.0 (39.3–68.2) | 15/50 | 30.0 (17.9–44.6) |
| Day 42 |  |  |  |  |  |  |  |  |
| Seronegative | 19/31 | 61.3 (42.2–78.2) | 8/31 | 25.8 (11.9–44.6) | 14/28 | 50.0 (30.6–69.4) | 8/28 | 28.6 (13.2–48.7) |
| Seropositive | 4/18 | 22.2 (6.4–47.6) | 2/18 | 11.1 (1.4–34.7) | 13/21 | 61.9 (38.4–81.9) | 3/21 | 14.3 (3.0–36.3) |
| All | 23/49 | 46.9 (32.5–61.7) | 10/49 | 20.4 (10.2–34.3) | 27/49 | 55.1 (40.2–69.3) | 11/49 | 22.4 (11.8–36.6) |
|  |  |  |  |  |  |  |  |  |
| **ADCC antibodies** | (4-fold increase) | | (16-fold increase) | | (4-fold increase) | | (16-fold increase) | |
|  | n/N^c^ | % (95% CIs) | n/N^c^ | % (95% CIs) | n/N^c^ | % (95% CIs) | n/N^c^ | % (95% CIs) |
| **A/California/7/2009 strain** |  |  |  |  |  |  |  |  |
| Day 21 |  |  |  |  |  |  |  |  |
| Seronegative | 1/2 | 50.0 (1.3–98.7) | 1/2 | 50.0 (1.3–98.7) | 3/3 | 100 (29.2–100) | 3/3 | 100 (29.2–100) |
| Seropositive | 20/35 | 57.1 (39.4–73.7) | 7/35 | 20.0 (8.4–36.9) | 21/34 | 61.8 (43.6–77.8) | 9/34 | 26.5 (12.9–44.4) |
| All | 21/37 | 56.8 (39.5–72.9) | 8/37 | 21.6 (9.8–38.2) | 24/37 | 64.9 (47.5–79.8) | 12/37 | 32.4 (18.0–49.8) |
| Day 42 |  |  |  |  |  |  |  |  |
| Seronegative | 3/3 | 100 (29.2–100) | 3/3 | 100 (29.2–100) | 3/3 | 100 (29.2–100) | 2/3 | 66.7 (9.4–99.2) |
| Seropositive | 22/34 | 64.7 (46.5–80.3) | 11/34 | 32.4 (17.4–50.5) | 20/32 | 62.5 (43.7–78.9) | 10/32 | 31.3 (16.1–50.0) |
| All | 25/37 | 67.6 (50.2–82.0) | 14/37 | 37.8 (22.5–55.2) | 23/35 | 65.7 (47.8–80.9) | 12/35 | 34.3 (19.1–52.2) |
| **A/Brisbane/59/2007 strain** |  |  |  |  |  |  |  |  |
| Day 21 |  |  |  |  |  |  |  |  |
| Seronegative | 0/1 | 0.0 (0.0–97.5) | 0/1 | 0.0 (0.0–97.5) | 3/4 | 75 (19.4–99.4) | 2/4 | 50 (6.8–93.2) |
| Seropositive | 20/28 | 71.4 (51.3–86.8) | 10/28 | 35.7 (18.6–55.9) | 22/32 | 68.8 (50.0–83.9) | 15/32 | 46.9 (29.1–65.3) |
| All | 20/29 | 69.0 (49.2–84.7) | 10/29 | 34.5 (17.9–54.3) | 25/36 | 69.4 (51.9–83.7) | 17/36 | 47.2 (30.4–64.5) |
| Day 42 |  |  |  |  |  |  |  |  |
| Seronegative | 0/1 | 0.0 (0.0–97.5) | 0/1 | 0.0 (0.0–97.5) | 2/3 | 66.7 (9.4–99.2) | 2/3 | 66.7 (9.4–99.2) |
| Seropositive | 17/30 | 56.7 (37.4–74.5) | 13/30 | 43.3 (25.5–62.6) | 23/32 | 71.9 (53.3–86.3) | 13/32 | 40.6 (23.7–59.4) |
| All | 17/31 | 54.8 (36.0–72.7) | 13/31 | 41.9 (24.5–60.9) | 25/35 | 71.4 (53.7–85.4) | 15/35 | 42.9 (26.3–60.6) |

Abbreviations: ADCC, antibody-dependent cell-mediated cytotoxicity; AS03, Adjuvant System containing DL-α-tocopherol and squalene in an oil-in-water emulsion; CDL, complement dependent lysis; CI, confidence interval.

^a^ For CDL antibodies, vaccine response in seropositive subjects was defined as a ≥3-fold increase or ≥9-fold increase over pre-vaccination baseline levels, each applying at Day 21 and at Day 42; for seronegative subjects with baseline titers of <32.0 1/DIL, post-vaccination reciprocal titers of ≥96.0 1/DIL or ≥288.0 1/DIL were required to meet these 3-fold and 9-fold thresholds respectively.

^b^ For ADCC antibodies, vaccine response was defined as a ≥4-fold increase or ≥16-fold increase over pre-vaccination baseline levels, each applying at Day 21 and at Day 42; for seronegative subjects with baseline titers of <32.0 1/DIL, post-vaccination reciprocal titers of ≥128 1/DIL or ≥512.0 1/DIL were required to meet these 4-fold and 16-fold thresholds respectively.

^c^ n = number of subjects with vaccine response; N = number of subjects with available results.

**SUPPLEMENTARY TABLE 2.** Study group vaccine response (VR) comparisons (according-to-protocol cohort for immunogenicity)

|  | **Between-group difference**  **(AS03-adjuvanted vaccine – non-adjuvanted vaccine) in percentage of subjects with VR** | | | |
| --- | --- | --- | --- | --- |
|  | **CDL antibody response**  **% (95% CIs)^a^** | | **ADCC antibody response**  **% (95% CIs)^b^** | |
| **Vaccine response (Day 42)** | (3-fold increase) | (9-fold increase) | (4-fold increase) | (16-fold increase) |
| **A/California/7/2009** |  |  |  |  |
| Seronegative^c^ | 9.7 (−2.1; 25.1) | 9.7 (−2.1; 25.1) | 0.0 (−60.6; 60.6) | −33.3 (−81.5; 40.3) |
| Seropositive | 11.1 (−8.8; 33.2) | 10.5 (−16.6; 36.5) | −2.2 (−25.1; 20.7) | −1.1 (−23.3; 21.4) |
| All | 10.2 (2.4; 21.8) | 10.1 (−1.8; 23.3) | −1.9 (−23.5; 19.8) | −3.6 (−25.2; 18.6) |
| **A/Brisbane/59/2007** |  |  |  |  |
| Seronegative^c^ | −11.3 (−35.3; 14.0) | 2.8 (-19.9;25.7) | 66.7 (−45.2; 95.0) | 66.7 (−45.2; 95.0) |
| Seropositive | 39.7 (8.3; 63.7) | 3.2 (−21.4; 26.2) | 15.2 (−8.8; 37.8) | −2.7 (−26.7; 21.5) |
| All | 8.2 (−11.6; 27.3) | 2.0 (−14.5; 18.6) | 16.6 (−6.8; 38.6) | 0.9 (−22.7; 24.2) |

Abbreviations: ADCC, antibody-dependent cell-mediated cytotoxicity; AS03, Adjuvant System containing DL-α-tocopherol and squalene in an oil-in-water emulsion; CDL, complement dependent lysis; CIs, confidence intervals.

^a^ For CDL antibodies, vaccine response was defined as a ≥3-fold increase or ≥9-fold increase over pre-vaccination baseline levels.

^b^ For ADCC antibodies, vaccine response was defined as a ≥4-fold increase or ≥16-fold increase over pre-vaccination baseline levels.

^c^ Seronegative subjects are those with CDL or ADCC antibody titers <32 1/DIL against A/California/7/2009 and A/Brisbane/59/2007 H1N1 strains prior to vaccination.

**SUPPLEMENTARY TABLE 3.** Mean geometric increases (MGIs), adjusted geometric mean titers (GMTs) and adjusted-group ratios (according-to-protocol cohort for immunogenicity).

|  | **Non-adjuvanted vaccine (n=50)** | | **AS03-adjuvanted vaccine (n=52)** | |  |
| --- | --- | --- | --- | --- | --- |
|  | **MGI (95% CIs)^a^** | **Adjusted GMT** | **MGI (95% CIs)^a^** | **Adjusted GMT** | **Adjusted-GMT ratio**  **AS03-adjuvanted vaccine / non-adjuvanted vaccine**  **(95% CIs)^b^** |
| **CDL antibodies** |  |  |  |  |  |
| **A/California/7/2009** |  |  |  |  |  |
| Day 21 | 54.1 (31.3–93.7) |  | 118.2 (76.5–182.4) |  |  |
| Day 42 | 60.6 (35.6–103.1) | 1679.1 | 217.0 (135.5–347.5) | 5332.2 | 3.2 (1.8–5.5) |
| **A/Brisbane/59/2007** |  |  |  |  |  |
| Day 21 | 2.6 (1.6–4.2) |  | 5.0 (3.1–8.1) |  |  |
| Day 42 | 4.5 (2.6–7.8) | 132.2 | 5.5 (3.6–8.4) | 172.2 | 1.3 (0.7–2.5) |
| **ADCC antibodies** |  |  |  |  |  |
| **A/California/7/2009** |  |  |  |  |  |
| Day 21 | 5.4 (2.9–10.1) |  | 7.2 (3.1–16.6) |  |  |
| Day 42 | 9.8 (5.1–18.9) | 11466.9 | 9.0 (3.8–21.0) | 9566.1 | 0.8 (0.3–2.1) |
| **A/Brisbane/59/2007** |  |  |  |  |  |
| Day 21 | 8.0 (4.2–15.3) |  | 13.5 (6.4–28.5) |  |  |
| Day 42 | 7.5 (3.9–14.5) | 8198.9 | 10.8 (5.6–20.7) | 10673.4 | 1.3 (0.6–3.1) |

Abbreviations: ADCC, antibody-dependent cell-mediated cytotoxicity; AS03, Adjuvant System containing DL-α-tocopherol and squalene in an oil-in-water emulsion; CDL, complement dependent lysis; CIs, confidence intervals.

^a^ MGI was defined as the geometric mean fold rise in GMTs at Day 21/Day 42 relative to Day 0 (and derived from the Day 21/Day 42 GMT: Day 0 GMT ratio).

^b^ For between-group comparisons, adjusted-GMT ratios at Day 42 were estimated via Analysis of Covariance (ANCOVA) models, including the vaccine group as a fixed effect and age and the baseline value (Day 0) as covariates.

**SUPPLEMENTARY TABLE 4** Hemagglutination inhibition (HI), complement-dependent lysis (CDL), and antibody-dependent cell-mediated cytotoxicity (ADCC) antibody titer correlations at Day 42 (according to protocol cohort for immunogenicity).

|  | **Non-adjuvanted vaccine** | **AS03-adjuvanted vaccine** | **All subjects** |
| --- | --- | --- | --- |
| **HI-CDL** |  |  |  |
| A/California/7/2009 strain | r=0.69 | r=0.68 | r=0.70 |
| A/Brisbane/59/2007 strain | r=0.26 | r=0.91 (r=0.14)^a^ | r=0.84 (r=0.17)^a^ |
| **HI-ADCC** |  |  |  |
| A/California/7/2009 strain | r=0.44 | r=0.26 | r=0.35 |
| A/Brisbane/59/2007 strain | r=0.26 | r=0.26 (r=0.24)^a^ | r=0.24 (r=0.26)^a^ |
| **CDL-ADCC** |  |  |  |
| A/California/7/2009 strain | r=0.48 | r=0.24 | r=0.35 |
| A/Brisbane/59/2007 strain | r=0.10 | r=0.26 (r=0.22)^a^ | r=0.17 (r=0.10)^a^ |

Abbreviations: AS03, Adjuvant System containing DL-α-tocopherol and squalene in an oil-in-water emulsion; r, correlation coefficient (linear regression fit).

^a^ Adjusted by removal of an outlier (patient #647).

**FIGURE S1** Reverse cumulative distribution curves for antibody responses against influenza strains (according-to-protocol cohort for immunogenicity).


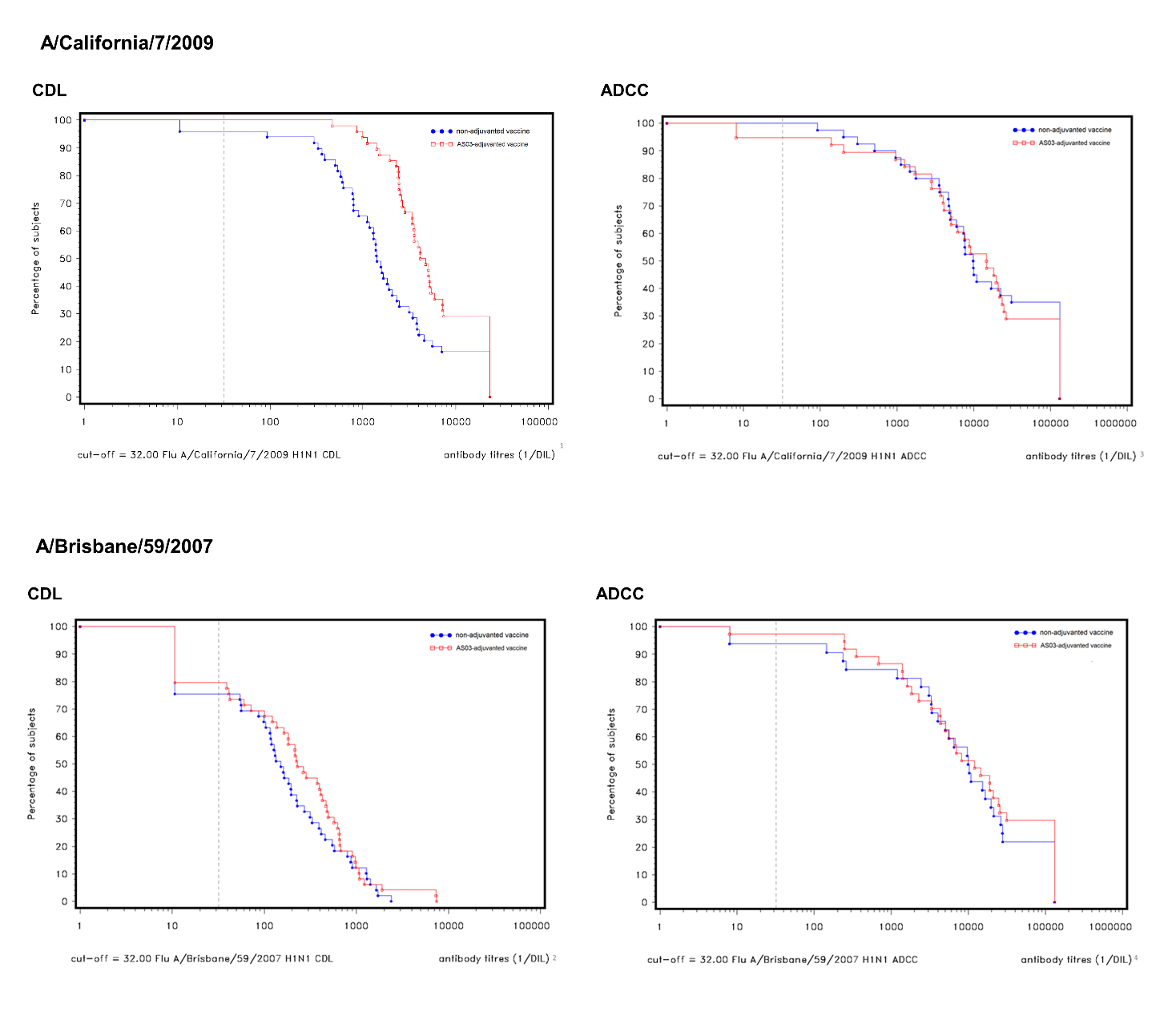


Reverse cumulative distribution curves demonstrating responses against A/California/7/2009 and A/Brisbane/59/2007 strains at Day 42 (21 days after the second doses of non-adjuvanted [blue] or AS03-adjuvanted [red] A(H1N1)pdm09 vaccine).

Abbreviations: ADCC, Antibody-dependent cell-mediated cytotoxicity responses; CDL, Complement-dependent lysis responses.

**FIGURE S2** Correlations between hemagglutination inhibition (HI) and complement-dependent lysis (CDL) antibody titers against A/California/7/2009 vaccine strain at Day 42.


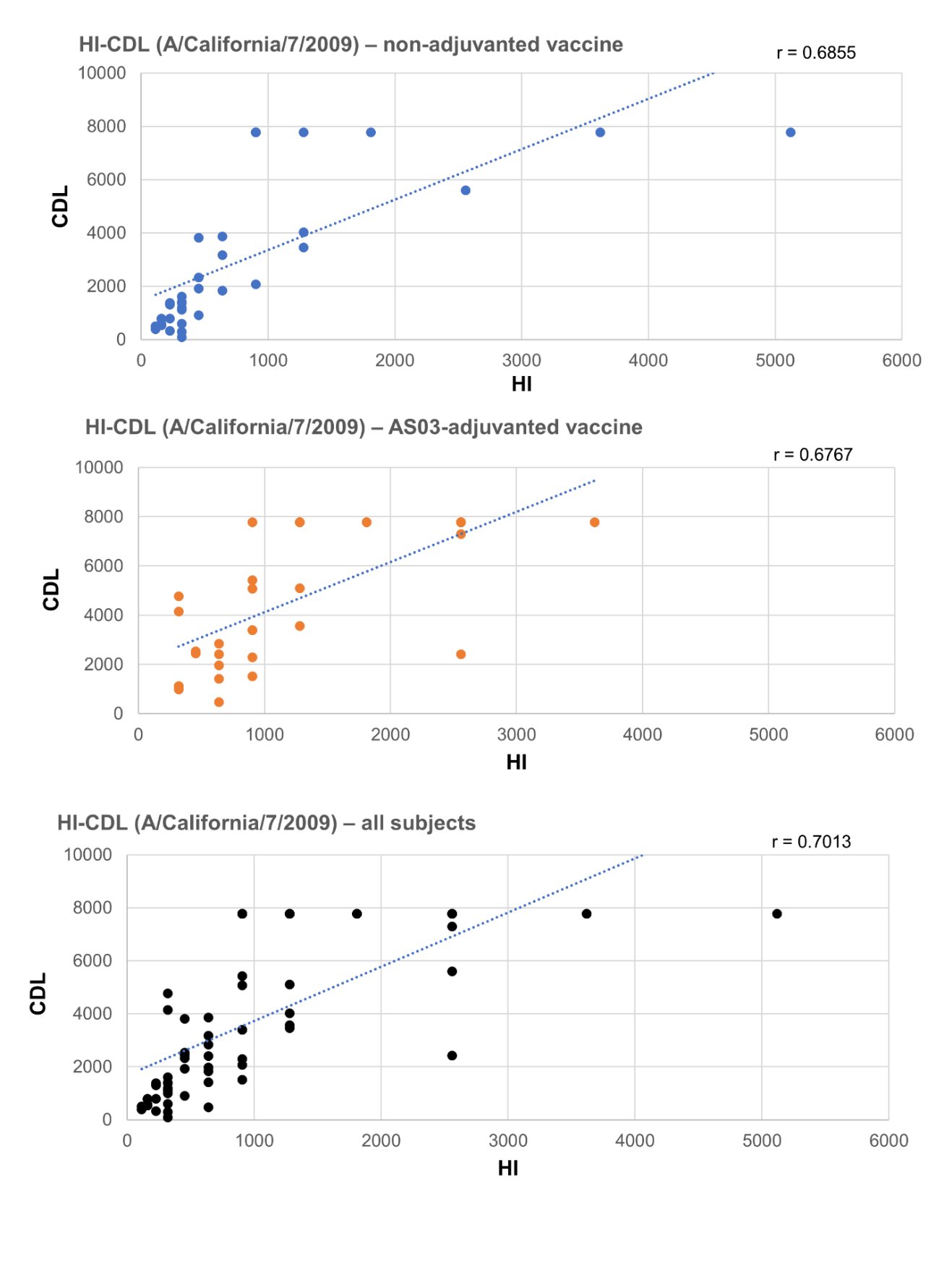


Abbreviations: AS03, Adjuvant System containing DL-α-tocopherol and squalene in an oil-in-water emulsion; r, correlation coefficient.
